# BioNexusSentinel: a visual tool for bioregulatory network and cytohistological RNA-seq genetic expression profiling within the context of multicellular simulation research using ChatGPT-augmented software engineering

**DOI:** 10.1093/bioadv/vbae046

**Published:** 2024-03-20

**Authors:** Richard Oliver Matzko, Savas Konur

**Affiliations:** School of Computer Science, AI and Electronics, University of Bradford, Bradford BD7 1HR, United Kingdom; School of Computer Science, AI and Electronics, University of Bradford, Bradford BD7 1HR, United Kingdom

## Abstract

**Summary:**

Motivated by the need to parameterize ongoing multicellular simulation research, this paper documents the culmination of a ChatGPT augmented software engineering cycle resulting in an integrated visual platform for efficient cytohistological RNA-seq and bioregulatory network exploration. As contrasted to other systems and synthetic biology tools, BioNexusSentinel was developed *de novo* to uniquely combine these features. Reactome served as the primary source of remotely accessible biological models, accessible using BioNexusSentinel’s novel search engine and REST API requests. The innovative, feature-rich gene expression profiler component was developed to enhance the exploratory experience for the researcher, culminating in the cytohistological RNA-seq explorer based on Human Protein Atlas data. A novel cytohistological classifier would be integrated via pre-processed analysis of the RNA-seq data via R statistical language, providing for useful analytical functionality and good performance for the end-user. Implications of the work span prospects for model orthogonality evaluations, gap identification in network modelling, prototyped automatic kinetics parameterization, and downstream simulation and cellular biological state analysis. This unique computational biology software engineering collaboration with generative natural language processing artificial intelligence was shown to enhance worker productivity, with evident benefits in terms of accelerating coding and machine-human intelligence transfer.

**Availability and implementation:**

BioNexusSentinel project releases, with corresponding data and installation instructions, are available at https://github.com/RichardMatzko/BioNexusSentinel.

## 1 Introduction

Multicellular simulation modelers, as well as machine learning (ML) practitioners, rely on parallelization using central processing unit (CPU), graphics processing unit (GPU), or tensor processing unit (TPU) hardware. However, powerful hardware and ML algorithms alone are insufficient for the task of replicating biochemical and biophysical multicellular reality. The task rests squarely upon the availability and processing of information (Perrakis and Sixma 2021). Computer-assisted design (CAD) is invariably data driven and it was suggested that tools predictive of biological behaviour would enhance CAD design in synthetic biology (Lux *et al.* 2011). There are medical implications regarding well parameterized multicellular simulations. For example, the complete generation of a tissue via a synthetic multicellular network had not been achieved (Santorelli *et al.* 2019); however, the engineering of multicellular self-organization was considered vital to next generation regenerative medicine. Multicellular simulations could be used to study and design for heterogeneous populations, morphogenesis and differentiation. However, perhaps besides the heavily funded Blue Brain Project (Markram 2006), multicellular simulation parameterization falls short. The BioNexusSentinel (BNS) platform was developed to investigate ways to address this problem. It would expand on classical open source synthetic biology (Chandran and Sauro 2012, Watanabe *et al.* 2019, Konur *et al.* 2021) and systems biology tools (COPASI 2022) by providing unified and rapid accessibility to an end-user simultaneously for downloadable multispecies bioregulatory network models and RNA expression data on a cytohistological basis. These features were envisioned as useful for the parameterization of future multicellular simulation systems, for instance the use of signalling or metabolic network models, or for connecting to ground truth cell specific data for differential genetic molecular states.

By connecting to bioinformatics resources such as Reactome (Büchel *et al.* 2013), BNS could operate as a network exploration tool, whilst also being coupled to RNA-seq expression data from the Human Protein Atlas (Human_Protein_Atlas 2022, Watson *et al.* 2022) (HPA). The work would demonstrate how RNA-seq expression data, network exploration and database connectivity could be integrated for future biological CAD. Whilst requiring additional research continuity, this endeavour provided a foundation upon which complex multimodality can be concretely, ergonomically and reproducibly integrated.

BNS presented novelty in the way that it combined informatics resources, tools and libraries, emphasizing an ergonomic, accessible, visual workflow; and offered an alternative template compared to other approaches (Watanabe *et al.* 2019, Konur *et al.* 2021, TinkerCell_Website 2022) directed towards integrated platforms for synthetic biology CAD, rare in emphasizing eukaryotic multicellularity, and in contrast to the synthetic biology parts (Chandran *et al.* 2010, Watanabe *et al.* 2019), or domain specific language emphasis (Konur *et al.* 2021) of other solutions. Although without an emphasis on genetic parts, identifying parts requires a means to explore regulatory systems, such as via BNS. Indeed, the expansion of the synthetic biology repertoire was suggested, for instance in the context of signalling pathways (Toda 2020). The utility of the present work is as diverse as multicellular model design, eukaryotic parts identification, and even the pursuit of therapeutic targets, not to mention potential use in education.

As evident from notable efforts (Kang *et al.* 2014, Naylor *et al.* 2017, Ghaffarizadeh *et al.* 2018, Li *et al.* 2019), multicellular simulation requires increased parameterization with experimental biological data, a consequence of being a highly demanding discipline spanning biology, computer science and physics. Consequently, the biological emphasis may be neglected due to these other requirements. This study investigated the potential to narrow that gap by combining biological network exploration with the development of a cytohistological genetic encyclopaedia. An objective would be to develop CAD solutions for the injection of biological data modules into spatiotemporal simulations, with vast biological design implications. Our ongoing research concerned the multiscale layering of biophysics with biochemical solvers (Matzko *et al.* 2023). The use of bioregulatory network models with kinetic dynamics written in SBML (Systems Biology Markup Language) are of particular interest for these purposes at the subcellular scale, which can be loaded into memory and executed beside biophysical solvers using high performance computing.

BNS would be developed to interact with various online data resources, emphasizing the Reactome database (Gillespie *et al.* 2022). Reactome contains a comprehensive collection of SBML models for a range of species including *Homo sapiens*. To the best of knowledge, no local software tool encountered for synthetic biology or systems biology interacted with this database through REST API, let alone for multicellular simulation purposes. BNS integrated network exploration of such models with visualization options, unique parameter exploration, ergonomic SBML download to disc, local file organization, search features, prototyped automatic SBML kinetic parameterization (useful for time-course simulations), and the unique coupling with genetic expression profiling capabilities. BNS could be used to universally profile SBML models, although designed for the structure of Reactome SBML files.

Technological opportunities continue to evolve, and this work demonstrated a collaboration between human and artificial intelligence (AI) in the ground up development of the BNS platform, harnessing the programmatically capable, conversational natural language processing (NLP) large language model (LLM) agents ChatGPT-3.5 and 4 by OpenAI (OpenAI 2023). These would be utilized for generative coding and concept exploration. Fully automated software development appears to be within reach, but the key is in the validation phase just as in any ML, with the inevitable need for specifications. In this work, NLP evidenced a capacity to boost software engineer productivity. However, not without the significant and time-consuming executive decisions, designs, creativity and validation of the human developer. A strong analogy would be of a very hands-on supervisor directing and correcting an extremely capable and insightful worker. Human validation would be needed to integrate, test and expand generated algorithmic functionality.

GPT in ChatGPT stands for Generative Pre-trained Transformer. The idea of a Transformer architecture was proposed that would use only multi-head attention rather than recurrence and convolutions for sequence modelling, and originated from Google (Vaswani *et al.* 2017). GPT-2 was a 1.5 billion parameter Transformer (Radford *et al.* 2019) trained on large datasets of web scrapes. GPT-3 was also a large language model (LLM) of 175 billion parameters, where larger models were associated with better use of context, zero-shot performance, few-shot performance and text synthesis (Brown *et al.* 2020). GPT-3 used the same architecture as GPT-2 except using alternating dense and locally banded sparse attention patterns, like a Sparse Transformer. GPT-3 comprised 96 layers and 96 attention heads, each attention head of dimension 128, although with known weaknesses in comparing sentences such as paraphrasing, different usages of the same word and arithmetic. Such models are capable of descrambling words, made possible due to Byte-Pair encoding (Vaswani *et al.* 2017). Indeed, these characteristics give insights into the limitations of such models in automatic coding, and help to explain why extensive iterative practices were required with debugging and functional validation in developing BNS. Naturally, the inner operations of the models were beyond the scope of this work, not only due to being proprietary, but also the sheer scale of the models and the potential hardware requirements, in addition to being ML black boxes. This work did not explore ChatGPT’s API features.

In the initial development cycle, the GPT-4 model was available with significant online restrictions, hence the GPT-3.5 model was used primarily. It could be envisaged that the amount of human validation would decrease as NLP/LLM agents gained improved architectures and training. Indeed, GPT-4 was considered more productive, nevertheless also requiring human executive guidance through careful prompting. ChatGPT could be extremely insightful and often contributed methods and concepts that the user did not prompt, or even know of. Assistance could be provided for small or quite large tasks, from syntactic corrections to developing large code blocks. For example, PathwayMapGenerator.cs in BNS consisted of almost 600 lines of ChatGPT generated code, including its insightful use of C# lists and custom programmatic objects for reaction, reactant, modifier and product lookups in order to build a network into memory from SBML parsing via regular expressions. Although the work described in this paper primarily used text-to-text synthesis in the development of BNS, ChatGPT models have continued to be incrementally expanded, such as offering the live search of websites and impressive image-to-text capabilities.

## 2 Methods and results

BNS was written primarily in WinForms for Windows in C# .NET, also experimentally executing external Python scripts. BNS is an experimental integrated platform for biochemical pathway exploration, SBML model analysis/simulation, and cytohistological gene expression profiling. BNS was written primarily for Reactome database (Gillespie *et al.* 2022) SBML model compatibility. Reactome was chosen as a curated and expansive online database with SBML model download and web services.

### 2.1 Workflow demonstration and example use case

Before detailing the specific features of BNS, this section provides an illustrative introduction to the types of workflow and use cases possible with BNS at the time of writing. The described workflow can be replicated using BNS from the GitHub (Matzko 2023). The diagrams feature illustrative collages of screenshot fragments from the user interface.

**Figure 1.**
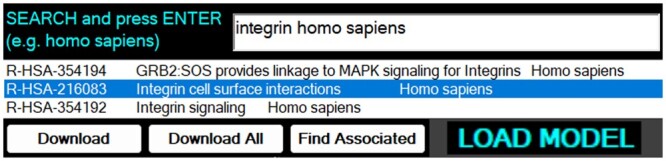
Use of the unique search engine to find and download SBML models, in addition to other options such as finding associated models and loading SBML models manually from disc. This collage was cropped to three results.

Figure 1 illustrates the use of the search engine, with integrin searched within the *Homo sapiens* species. Integrins are of interest from the multicellular simulation perspective given their role in the biophysical processes of adhesion and involvement in bioregulatory signalling via mechanotransduction, i.e. spatially and biochemically active. The R-HSA-216083 Reactome model was chosen. The download button would permit download of the selected SBML models, alternatively all models in the results list could be downloaded into a chosen folder in the file tree graph (not shown). The ‘Find Associated’ button provided the capacity to find associated models by reference to a pathway relations file obtained from Reactome. Alternatively, SBML models could be manually loaded via a ‘LOAD MODEL’ button. To date no issues were encountered with relation to parsing of custom SBML models; however, the algorithms were LLM generated and validated against Reactome models primarily. The model would thus be loaded into memory in a network structure utilizing object of object hierarchy, including lists of ‘reaction’ objects, generated via the PathwayMapGenerator C# file.

**Figure 2.**
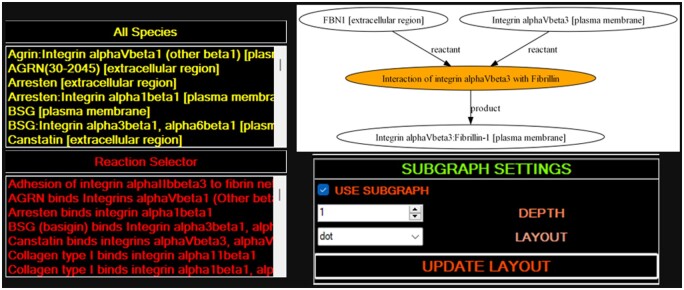
Important user interface controls on the Navigator menu upon loading the SBML model included molecular species and reaction list boxes, and the capacity to prune the network into ‘subgraphs’ at reaction ‘depths’ from the selected reaction.

Figure 2 illustrates how the integrin model that was downloaded and opened could be viewed in the main navigator of BNS, presenting with a list of species and reactions parsed from the model. This point in the workflow can still be improved, since finding the reactant of interest (FBN1) was achieved by right clicking the unpruned reaction map, which would open in the default image viewer. Once the reaction name was identified in this manual manner, the network could be pruned by using the ‘Reaction Selector’ and ‘SUBGRAPH SETTINGS’ in combination. Resulting diagrams are of publishable standard via GraphViz. From the network graph it could already be discerned that the reaction of interest involved a plasma membrane integrin interaction with an extracellular species, FBN1 (evidently a gene symbol), which would undergo an interaction to form a plasma membrane complex. Note the downstream truncation of this potential cascade, which is an identified limitation of using Reactome models. Within BNS non-orthogonality/orthogonality network overlap analysis was prototyped, but requires further research to validate and render most effective.

**Figure 3.**
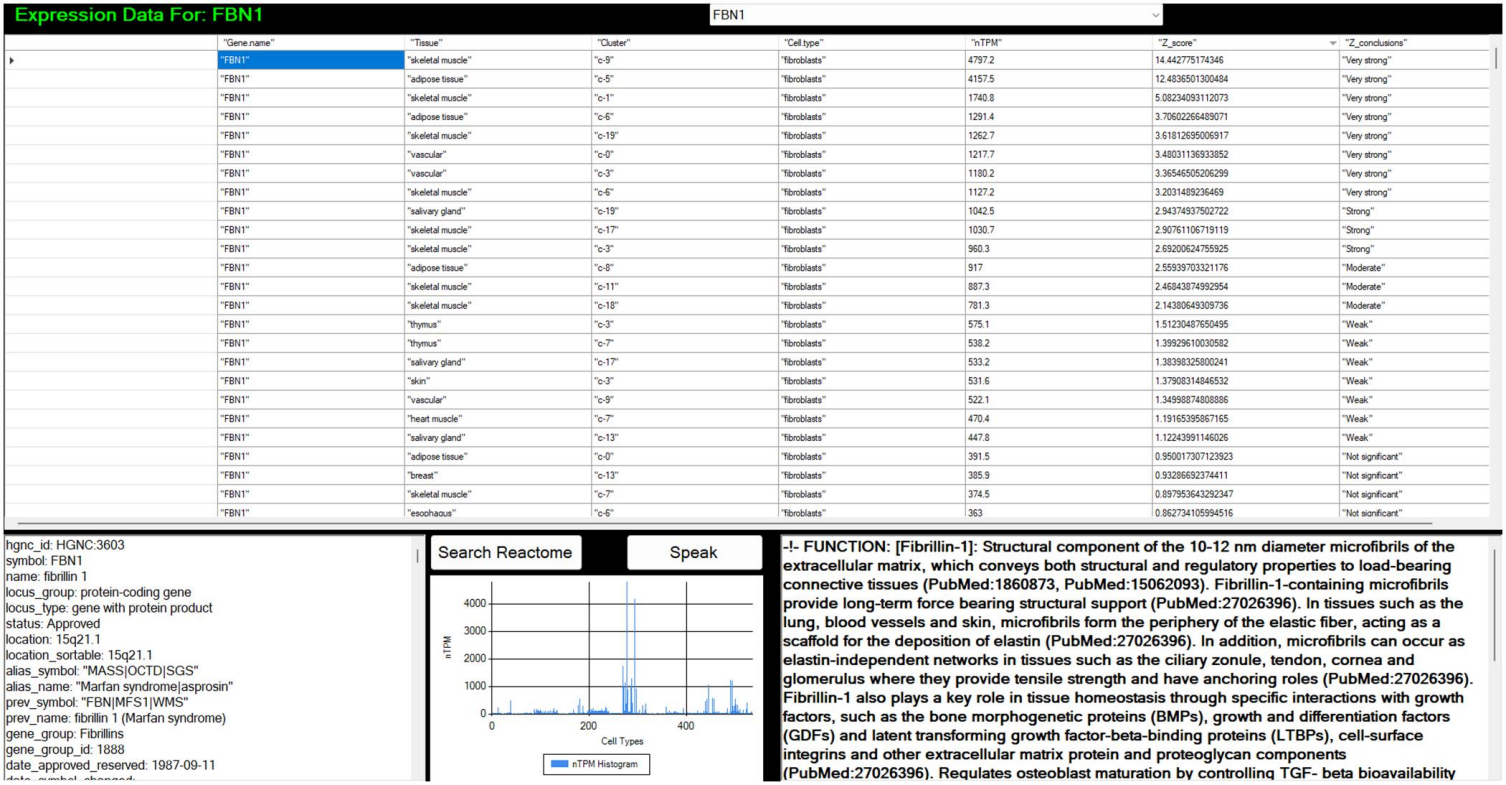
A screenshot of the GeneExpressionProfiler results window, in this case assessing cytohistological gene expression levels for FBN1. Unsurprisingly, Fibrillin-1 was expressed most highly in fibroblasts across various tissue types. Whilst the aesthetics have been improved in various ways, including accessibility with a text-to-speech capability, the overall layout can of course be subject to change. A histogram of gene expression distributions across the cell types is also presented in the lower middle of the screen. The descriptions represent an obvious target for NLP manipulations or chatbots in the future.

Having identified a gene of interest, the BNS GeneExpressionProfile (GEP) window could be used to query for the FBN1 gene, as in Fig. 3. Subsequently, differential expression analysis is displayed for different human cell lineages for this gene, along with additional information. Hence, it could be discerned that FBN1 represented Fibrilin-1 gene, an extracellular matrix component, providing context to the SBML reaction network, including with descriptions and other useful metadata, for instance disease associations and various identifiers. From Fig. 2, clicking a species would provide a popup window allowing the user to navigate and see links associated with the species from the SBML file. Using this, the gene symbol from UniProt could be determined for integrin alpha-V beta 3, ITGB3, and via GEP could be identified as an integrin with R-G-D amino acid sequence recognition, with a range of ligands, notable in platelet aggregation and with a very strong RNA-seq statistical expression level significance in platelets compared to any other cell.

### 2.2 BioNexusSentinel navigator

Requiring independent installation, GraphViz was previously used for interactive assembly trees for genetic constructs (Linshiz *et al.* 2016). BNS used GraphViz via DOT language generation from a model in memory parsed from SBML. Microsoft Automatic Graph Layout appeared antiquated, with GraphViz offering a more attractive network graphing solution.

Visualization of complex graphs with systems biology tools can be daunting with mixed results, and BNS strove to provide a presentable interface with ease of use. VisANT 5.0 is an attractive graphing tool (Granger *et al.* 2016) for plotting nodes and edges. However, BNS presents with a prioritized, seamless workflow, an ergonomic interface, multiple visualization methods and automatic logical format conversions to ensure compatibility with its components or for output.

**Figure 4.**
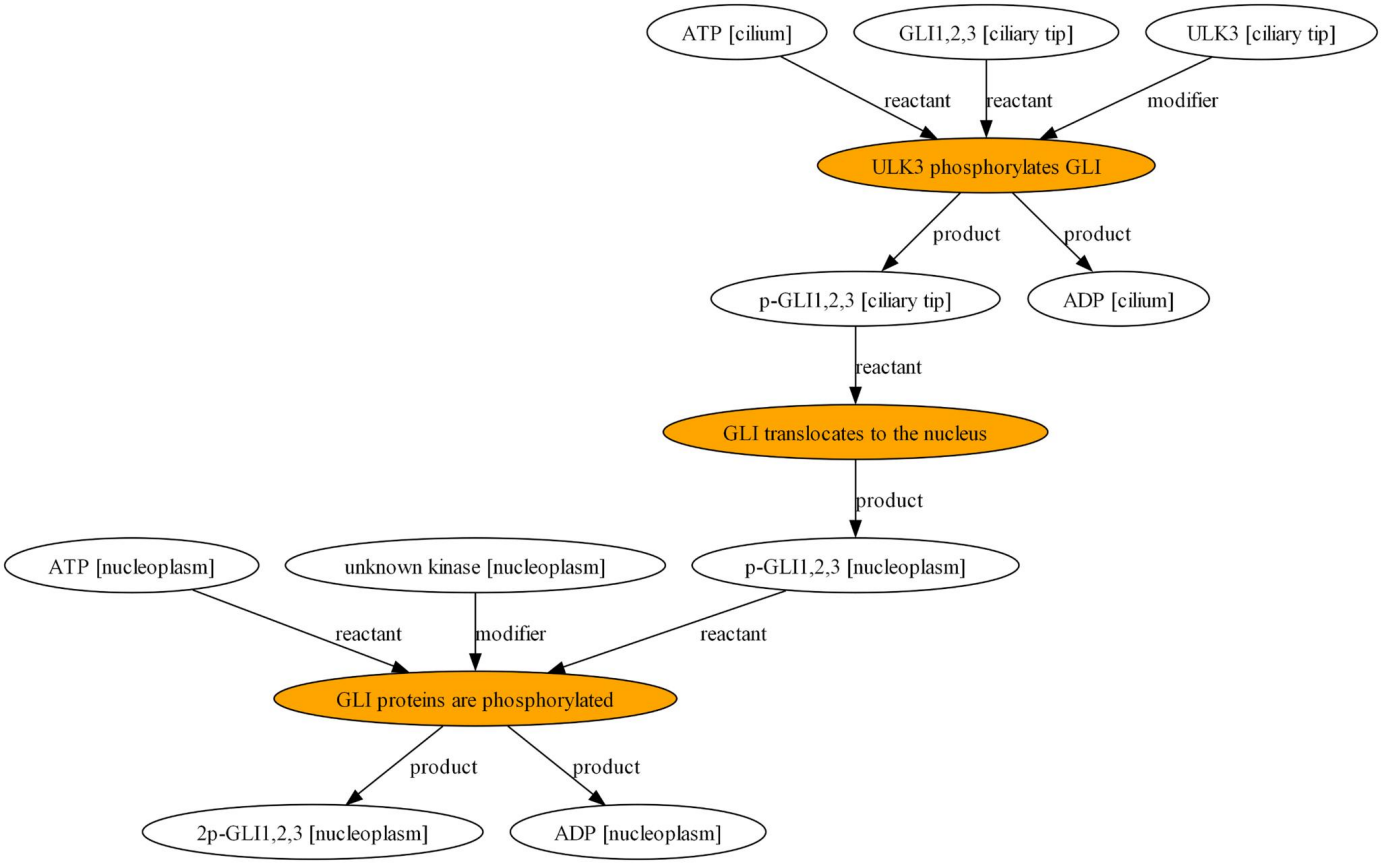
A GraphViz output via BioNexusSentinel with ‘depth’ 1 pruning around the ‘GLI translocates to the nucleus’ reaction of Reactome model R-HSA-5358351 for Signalling by Hedgehog. Depth 1 signifies pruning to 1 reaction distance. Reaction nodes are filled orange. Edges are labelled according to the types of reaction participants.

Modular design strategies are integral to synthetic biology design CAD and biological modelling. For example, the parts based approaches of synthetic biology or VisANT 5.0 (Granger *et al.* 2016) representing ecosystems by associating multiple models. The implication was that Reactome models might undergo such treatment not unlike genome-scale reconstructions (Büchel *et al.* 2013). This might be achieved by exploiting chemical species overlaps since individual Reactome models are biochemical network fragments. Whilst BNS did not harness expandable metanodes (Granger *et al.* 2016), BNS possessed a node pruning feature to allow centring on a selected reaction in the network (Fig. 4). Right click on the graph opened the file with default image viewer for further inspection. The output files would be saved to the executable directory, allowing for usage in publications.

**Figure 5.**
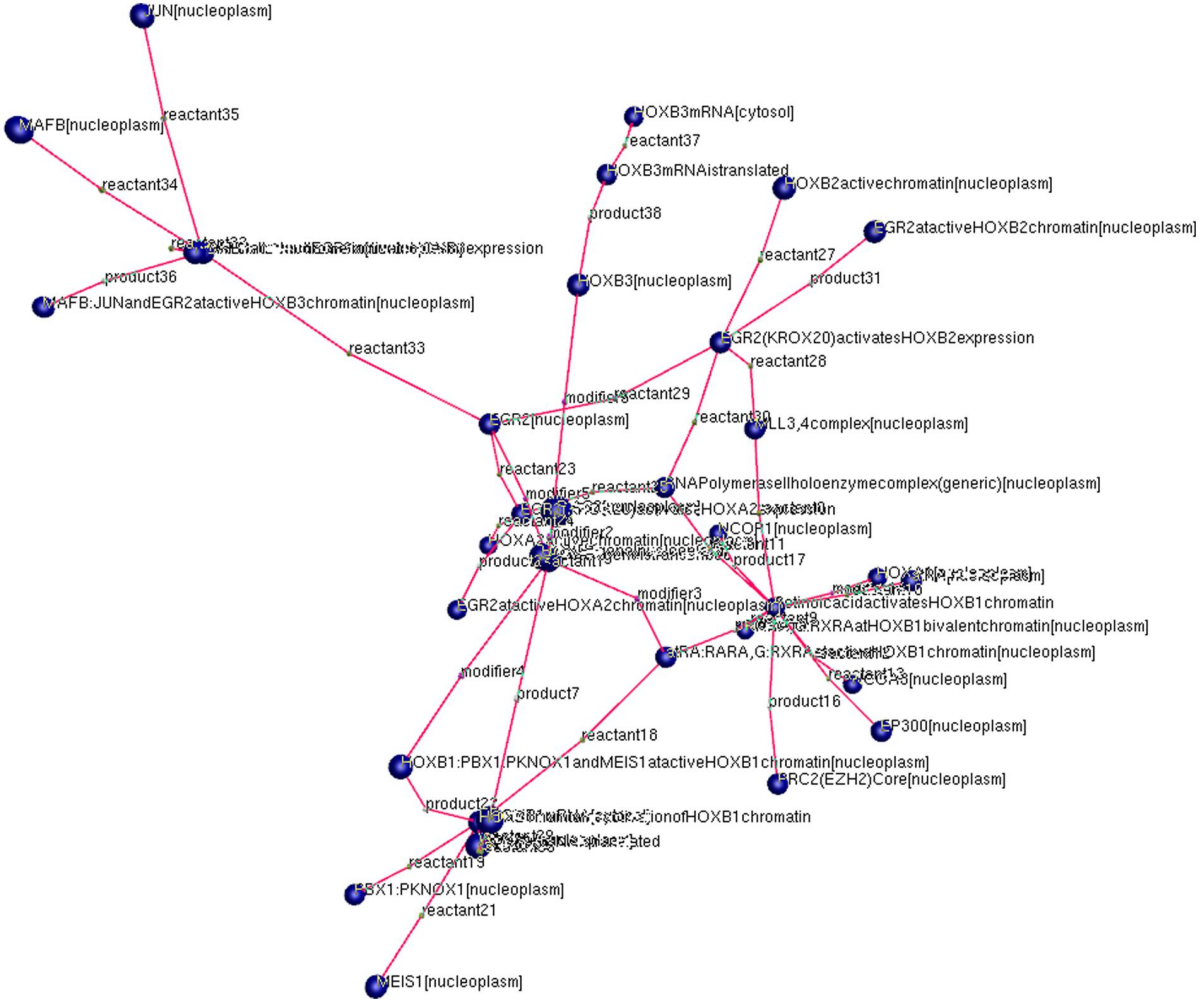
BioLayout 3.4 as launched by BioNexusSentinel via SIF files generated from a network representation in memory as loaded from SBML.

BNS could convert SBML files to .SIF format, automatically launched from BNS via BioLayout (Theocharidis *et al.* 2009) 3.4, requiring independent installation. BioLayout 3.4 provided for 3D network visualization capabilities (Fig. 5), with user directed pruning applied to the consequent SIF file.

Optical character recognition (OCR) was trialled using Tesseract 5, an LSTM (Long Short-Term Memory) neural network solution, obtained via NUGET for C#, which might have been useful for interactivity and visualization. OCR has been used in the past for biological datamining (Baltoumas *et al.* 2021). The pursuit of more reliable usage of machine learned models for this purpose could ensue. Given the use of a .NET C# interface for BNS, it would be possible to integrate Python script modules to augment BNS functionality in the future. This is achievable via Systems.Diagnosis.Process and System.IO.StreamWriter .NET classes for launching and feedback with Python scripts.

### 2.3 Model orthogonality and detecting bioinformatics gaps

**Figure 6.**
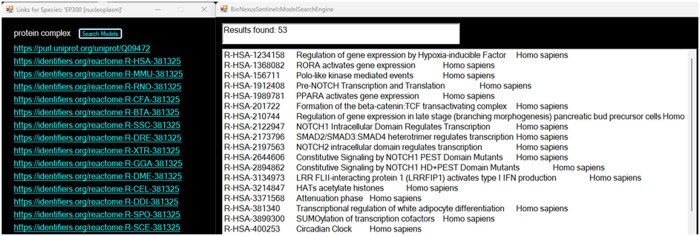
From the main BioNexusSentinel navigator, a species list extracted automatically from the SBML model allowed for selection of individual species, which resulted in the generation of an SBO identification (protein complex in this case), species identifier links if available and a ‘Search Models’ button (left). Upon clicking ‘Search Models’ the Model Search Engine dialog is opened, presenting with a list of models that are associated with the species identifiers according to the Reactome web services. In the future, BioNexusSentinel might allow species searches independently from loaded models.

Orthogonality refers to the isolation of interactions between system components, which can be synthetically modulated biologically (Miyamoto *et al.* 2013) and can manifest through unique genetic products (Jones *et al.* 2022). Model analytics can aid the assessment of orthogonality. BNS provided the capability to assess connected models in a few clicks. Once a compatible model is loaded, molecular species could be selected. Selection would generate a menu yielding the SBO (Systems Biology Ontology) species type and external reference links. Protein complexes possessed links for each species within the complex. The SBO label was retrieved using the SBO term parsed from the SBML using a local OWL (Web Ontology Language) file obtained from the EBI-BioModels GitHub (EBI-Biomodels 2023). A button in the menu listed Reactome models associated with the species within the ModelSearchEngine screen (Fig. 6). This function queried the Reactome content services using Uniprot identifiers or Gene Symbols via HTTP to return a string array of pathway interactions of the species. The extraction of identifiers and symbols was achieved through parsing of the available links embedded in the Reactome SBML model code by using regular expressions, e.g. string uniprotID = Regex.Match(link, @”[^/]+$”). Value. A class called HGNCMapper was created to map Ensembl identifiers to Gene Symbols. Indeed, gene identifiers are not used in a singular fashion across the genetics data community. This mapping would be achieved offline through the use of hgnc_complete_set_2023-04–01.csv, downloaded courtesy of EMBL’s European Bioinformatics Institute (EMBL-EBI 2023), as redirected from the HUGO Gene Nomenclature Committee (HGNC) (HGNC 2023). Ontological types not proteins or genes, such as small molecules, were not yet mapped to models, although external links were still presented in the menu.

The above capabilities provided for a step towards autonomous orthogonality analysis and the capability to manually assess pathway interactions via specific species. This might have relevance if a species operates as an input or output of the system; and could even result in the identification of gaps in the modelling of certain species. For example, the system output HOXD4 [HOXD4 (nucleoplasm)] of Reactome model R-HSA-5619507.sbml, which is a transcription factor (UniProt P09016), appears only as a product of this model and, apparently, had no appearances in other models. This implied that the vital actions of the transcription factor remained absent from the Reactome database. Hence, BNS was shown to provide for orthogonality assessment and functional gap identification of Reactome, where functional gaps reduce the capacity to interpret signals as kinetic relations for biophysical outcomes via bioregulatory mechanisms. Upon detection of such gaps, which might eventually be automatically reported, researchers can evaluate literature or assess connected models for the mechanisms of a given pathway. Design of experiments might then ensue to resolve any gaps in knowledge. Besides such gaps, Reactome was found to be incorrect in certain cases related to SBO code, e.g. model R-HSA-452723.sbml for DKK1 gene [nucleoplasm], ascribing it SBO : 0000297 (Protein Complex) incorrectly.

### 2.4 NetworkTools

**Figure 7.**
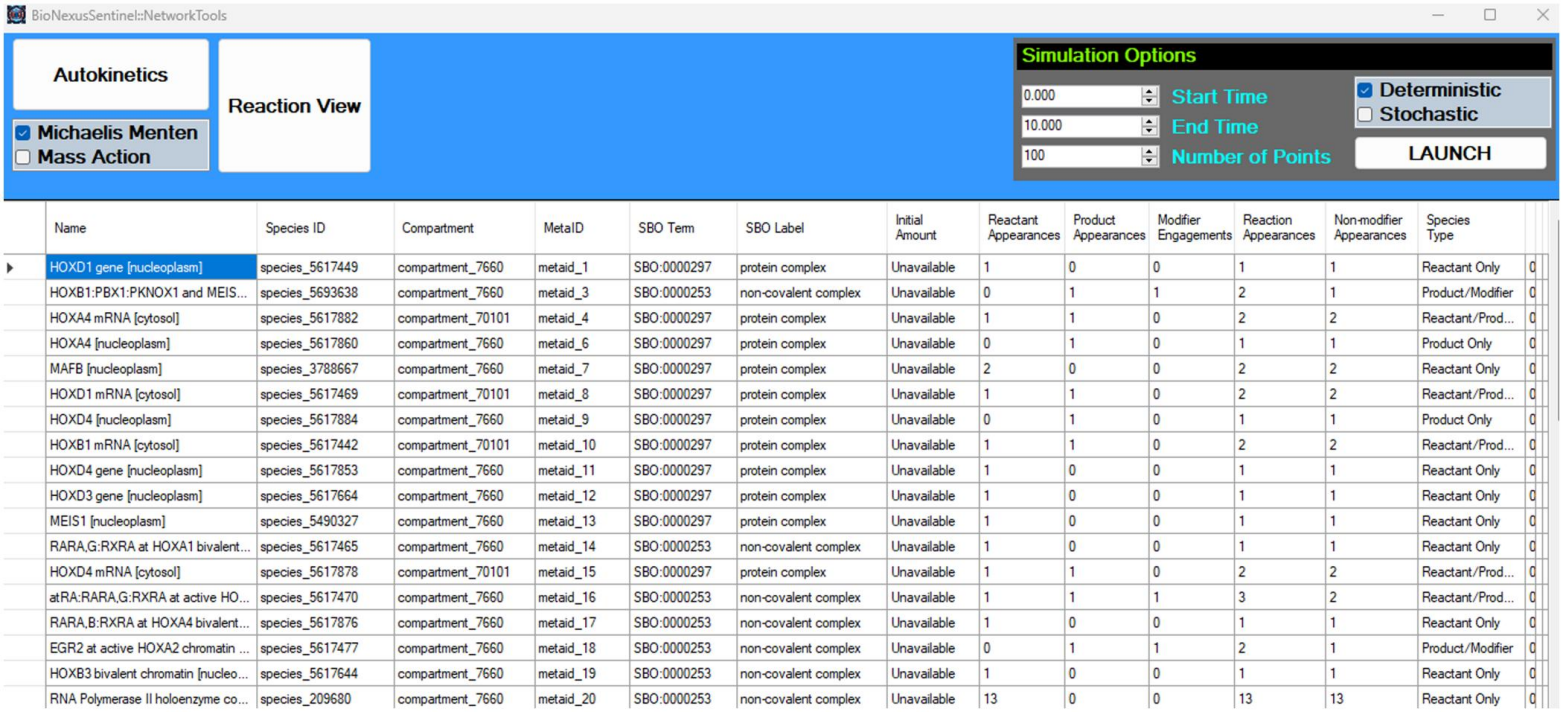
BioNexusSentinel::NetworkTools screen of species insights from a loaded SBML model. Network tools would also provide for a reaction view, displaying reactant, product and modifier names corresponding to each reaction in the .NET dataGridView table. An SBML simulation engine could be run from the top right of the screen with engine parameters. The species view mode could display species’ engagements with the network and expose its role as a system mediator, input or product. System inputs and outputs were also detailed on the ‘Navigator’ interface on initial SBML loading.

BNS provided an interface called NetworkTools (Fig. 7) to explore the composition of, as well as manipulate and simulate, SBML models. The Autokinetics button in BNS was a prototype feature that, with further research, should allow a user to automate the accurate parameterization of SBML models kinetically. Parameterization would involve modification of the SBML files by adding kinetic laws to the syntax. Efforts to use the automatic kinetics parameterization tool SBML-Squeezer 2 (Dräger *et al.* 2015) ran into a variety of errors, with resultant SBML files demonstrating simulation incompatibility with the COPASI application (Hoops *et al.* 2006) and Tellurium (Choi *et al.* 2018). Further parameterization investigations are required.

Through inference and optimization methods, including ML, the parameterization of complex networks for kinetics might be achieved. The plausibility of making ML predictions for biological purposes is well established in protein structure (Callaway 2022), which naturally has implications for mechanistic and hence kinetics predictions. BNS emphasized the use of the Reactome database (Gillespie *et al.* 2022) for SBML models. However, Reactome models are not kinetically parameterized and often involve enzymatic and higher order reaction configurations comprising multiple reactants and modifier species to generate products. Such reactions are often solved deterministically, for example through Hill functions in cooperative reactions, or through Michaelis Menten in other cases, as can be observed in reaction kinetics provided by SABIO-RK (Rojas *et al.* 2007).

Within NetworkTools, kinetically parameterized SBML models can be simulated. Tellurium (Choi *et al.* 2018) was the most successful means of simulation trialled with BioNexusSentinel, and was run with parameters via an external Python script from the C# code. Tellurium includes deterministic and stochastic simulation methods via libRoadRunner. The time-course results would be automatically launched in the default operating system browser via Plotly Open Source Graphing Library for Python. This exposed interpretation issues between the changes made by Autokinetics and the Tellurium simulation related to the time dimension of the modified SBML that would need to be resolved in the future. SBML kinetics models downloaded from SABIO-RK (Rojas *et al.* 2007) had appropriate behaviour. Nevertheless, the Autokinetics feature was still able to generate simulation capable models that demonstrated, albeit inaccurate, dynamic simulations from Reactome models that previously could not be simulated at all. Autokinetics had two prototypes. The first added a mass action kineticLaw to every SBML reaction and the second applied a Michaelis Menten kineticLaw. Eventually, the kinetic laws should be appropriate to specific reactions, and for stochastic simulations a method for fragmenting the mechanism with propensities might be required. Use of Python-COPASI API was attempted, but without initial success. Python-COPASI API supports bindings for Python, C# and Java.

While tools such as Infobiotics Workbench (Konur *et al.* 2021) can provide access to a simulation engine and domain specific language for modelling kinetics, BNS has considerable advantages when it comes to network analytics and existing network exploration. Regarding model compatibility, the highly cited COPASI software could not load BioModels repository (EMBL-EBI 2022) MODEL1208280001_url.xml for integrin activation, for syntactic reasons; whereas, ultimately, BNS was capable. The ability to simulate models from BioModels repository is variable on the model architecture. But as noted above with COPASI, such incompatibilities with systems biology or synthetic biology software and imported models are far from uncommon. Appropriate exception handling, version compatibility and invariance is required where syntax of models might vary. This demonstrates the need for continued support of such software to provide the flexibility and adaptability against use cases. However, BNS may also possess rigidity in terms of parsing via regular expressions that may be reevaluated. Many software prefer to use libSBML (Keating *et al.* 2020) for SBML parsing, including COPASI (Hoops *et al.* 2006); however, its limitations would need to be assessed. EMBL-EBI try to enforce ‘MIRIAM compliance’ for distributable SBML models (EMBL-EBI 2022), reportedly using COPASI as a curation tool.

### 2.5 ModelSearchEngine

The ModelSearchEngine component of BNS provides a simple but effective search utility across Reactome models by querying a local reference (ReactomePathways.txt) in tab separated format containing columns for model identifier, model name/description and organism. The user search input is searched on all three criteria. For example, one might search ‘glycolysis’ or ‘glycolysis homo sapiens’ to prioritize more specific results. The utility could be improved, e.g. by filtering irrelevant search results; particularly useful for the ‘Download All’ functionality.

Working with models with encoded names can get disorganized very quickly, and BNS made progress towards standardizing the procedure. BNS provided a .NET treeView control to represent a default directory structure that BNS generated for storing models. Downloaded models are stored in the directory that was last selected and can be opened in the treeView via double-click. Loading the model closes ModelSearchEngine and launches the model from the Navigator screen for investigation. Improvements can still be made through iterative means, including improved metadata presentation to the user and the possibility of using other repositories to acquire models, e.g. KEGG (Kyoto Encyclopedia of Genes and Genomes), BioModels (EMBL-EBI 2022), or the use of genome-scale models such as Recon3D (Brunk *et al.* 2018). However, Recon3D has limitations, for example lacking homeobox transcription factors or any polypeptide chains (SBO: 0000252).

### 2.6 GeneExpressionProfiler

**Figure 8.**
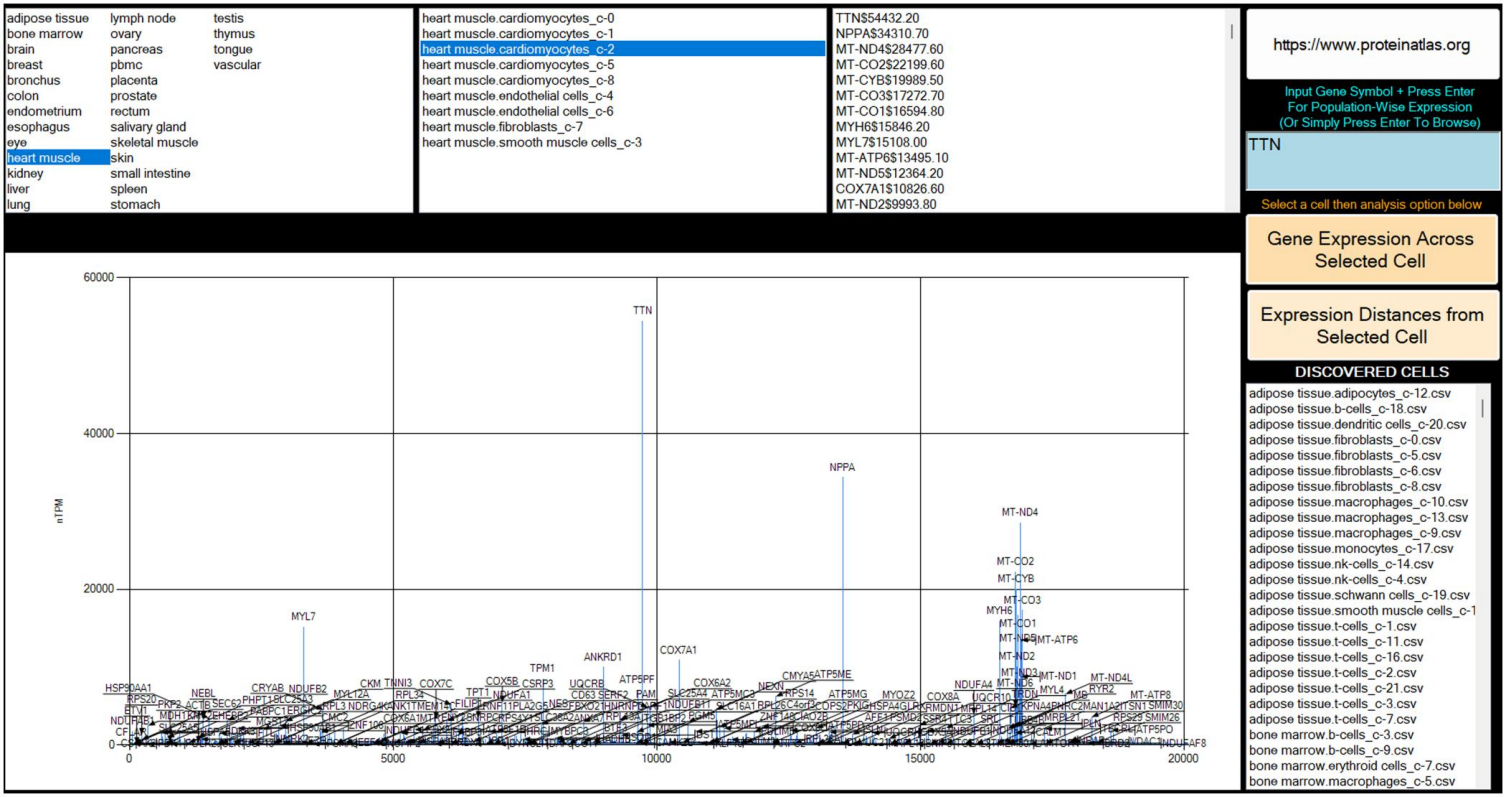
The recently updated GEP interface. Above, TTN (titin) RNA appeared highly expressed in heart muscle cardiomyocytes. Once searched, based on z-value thresholds it was found that this cell type had very high differential significance for this protein coding RNA, also very highly significant in skeletal myocytes. The high levels of NPPA natriuretic neuropeptide RNA expression were seen at only trace amounts in other tissues compared to heart muscle tissue, with very strong significance observed in cardiomyocytes.

BNS is not restricted to pathway investigations. The key motivation in developing BNS was to establish diverse phenotypic profiles based on bioregulatory veracity. To explore cellular diversity and function, a separate, novel project with a working title of GeneExpressionProfiler (GEP) was integrated in BNS (Fig. 8), accessible via the BioNexusSentinel::Navigator. Compared to other functions of BNS, this module was initially primarily entirely human coded. Subsequent vital updates augmented GEP with machine/human authored code, emphasizing pre-processed data. In the initial cycle, ChatGPT collaboration contributed in improving the description generator for genes by creating dictionary data containers using hgnc_complete_set_2023-04–01.csv (EMBL-EBI 2023), harvesting its descriptive data, and also using it to convert an input Gene Symbol to Uniprot (uniprotId) for an HttpClient request using the URL https://www.uniprot.org/uniprot/{uniprotId}.txt. Both sets of descriptive data could then be combined and displayed.

GEP would use cell specific RNA-seq data made available at the HPA (Human_Protein_Atlas 2022, Watson *et al.* 2022). The archive used, rna_single_cell_type_tissue.tsv, was selected as it possessed tissue origin and cell specific reads in nTPM (normalized transcripts per million). Data could thus be analysed and visualized for gene expression profiles on a tissue and cell specific basis across the ∼20 000 human genes. However, it was observed that cell types and tissues were still missing from the dataset, e.g. chondrocytes. All 13 protein coding mitochondrial genes appear to be accounted for in this dataset; however, the 22 tRNA and 2 rRNA genes are apparently not present. Neither apparently are non-coding genes, such as for non-coding RNAs (ncRNA), e.g. microRNAs (miRNAs). For instance, a search for SNORD115 ncRNA gene in GEP produced no results. MIR21 produced a description but no gene matches in the database. Indeed, the HPA was built on protein coding data (Digre and Lindskog 2023), and various Ensemble gene identifier searches with R were negative. This limitation can be investigated further. For example, the possible use of a manually curated database for miRNAs called MiRGeneDB (Fromm *et al.* 2022, MirGeneDB 2023), containing 16 670 evolutionarily classified miRNAs. miRNAs are considered important for post-transcriptional gene regulation. GeneCaRNA is an alternative ncRNA repository (GeneCards 2023). Reactome possesses a single model for miRNA biogenesis. However, BioModels possesses some miRNA associated models.

A tentative interactome graph generator was also developed. This InteractomeVisualizer of GEP showed only parent to child node interactions via GraphViz, although other solutions show child–child interactions. The human interactions data was acquired via the human portal of the Rat Genome Database (Rat_Genome_Database 2023) and algorithmically interpreted. FIsInGene_122921_with_annotations.txt from Reactome might be an alternative from the functional interactions section in downloads (Reactome 2022).

### 2.7 Towards a cell lineage classifier

GEP was considered for significant extensibility, and R analytics would be used to further analyse the HPA rna_single_cell_type_tissue.tsv data. R is a well-developed language for statistics with a range of available libraries. Knowing the exact libraries or methods can be quite daunting, and once again with sufficient persistence from a researcher, brainstorming with conversational AI can help in identifying methods and producing code. It helps to be reasonably conversant in the domain; however, from the syntactic perspective AI can save enormous amounts of time.

A major difference between R and BNS is the ergonomic value. R is a programming domain requiring specialist skills, albeit AI can bridge the gap, whilst BNS can be seen as an endeavour towards a CAD tool for an end-user to parameterize systems. On the other hand, R proved extremely valuable for reproducibly generating pre-processed data for deployment due to its scripted handling of complex datasets. The reproducibility is critical for future dataset expansibility, allowing the BNS frontend to display pre-processed data with minimal computational delays.

rna_single_cell_type_tissue.tsv has headers of Gene (Ensemble ID), Gene.name (Gene Symbol), Tissue, Cluster, Cell.type, Read.count and nTPM. Cluster would be expected to refer to phenotypic expression variants of cells belonging to the same tissue and cell type. nTPM is to be favoured over reads as it involves a considered normalization approach. nTPM was found to have an R^2^ linear regression correlation of 0.21 with reads, hence weakly correlating.

Data preparation would remove superfluous columns and reorganize the data. Read.count would be removed in favour of using nTPM and the Tissue/Cell.type columns would be merged into a Tissue_CellType column, in order to reflect the cell lineage in a single dimension. The Ensemble ID column would also be removed as it is less descriptive than Gene Symbol. This would result in columns of Gene.name, nTPM and Tissue_CellType. However, some Tissue_CellType duplicates would exist for genes due to clusters (Table 1).

**Table 1.** A random sequential subset of the cleaned data frame consisting of almost 11 million rows.^a^

Gene.name	nTPM	Tissue_CellType
TSPAN6	62.3	Placenta smooth muscle cells
TSPAN6	3.3	Placenta t-cells
TSPAN6	19.3	Placenta smooth muscle cells
TSPAN6	50.1	Placenta endothelial cells
TSPAN6	44.9	Placenta fibroblasts
TSPAN6	11.7	Placenta mixed immune cells

a Since the original dataset included clusters, there are duplicates for Tissue_CellType. Hence, these duplicate rows would be merged via median despite significant variance. This strategy was later reverted towards retaining cluster types within GEP, as apparent in Fig. 8.

To analyse the variation in the duplicates, a new data frame was generated, merging the duplicate Tissue_CellType and matching Gene.name columns with a new column for nTPM_list containing a list of all nTPM expression levels, along with columns for standard deviation, coefficient of variation, mean and median. From the coefficient of variation, it could be seen that nTPM within a Tissue_CellType gene specific entry was 721% of mean for ACTL10 gene in brain excitatory neurons, whilst the standard deviation was highest for DEFA5 with 541 585 in small intestine undifferentiated cells. These cell specific DEFA5 nTPM values were 882, 5116.4, and 941 045. Clearly, nTPM values could vary drastically within a single cell classification, notably in undifferentiated cells. In many Tissue_CellType and gene combinations there was only a solitary datapoint, hence na.rm = TRUE could be set in R when calculating the mean nTPM standard deviation and coefficient of variation to ignore NA values in these cases where such metrics were unavailable. This yielded mean nTPM standard deviation of 22.5 and a mean nTPM coefficient of variation of 63.14% across the dataset for expression clusters.

The intention was to develop a lineage classifier based on a gene expression feature vector input, i.e. input of nTPM gene expression for all gene types to yield a computed Tissue_CellType classification. For simplicity, despite the considerable phenotypic variation evidenced by expression differences within individual Tissue_CellTypes, the median would be taken of the nTPM_list for the clusters to yield a single value for Tissue_CellTypes per gene, rather than the mean to account for skewness (later reverted to retain individual clusters within the classifier). That said, in biological systems it can be argued that outliers do not exist, rather critical phenotypic variations exist that determine state.

The data frame would be reduced to Gene.name, Tissue_CellType and nTPM_median columns. The data frame was pivoted and the column for nTPM removed to create a matrix of gene expression levels with rows of the Tissue_CellType followed by the feature vector of expression levels (Table 2). Constant and zero value columns would be removed, ie columns that contained all the same values or zeros. In retrospect this might have removed certain relevant non-zero columns, fortunately the only constant columns were zero valued. The 218 unique Tissue_CellTypes matched the number of unique folders generated by BNS in its prior method of data restructuring. Due to the limitations of sample sizes, here merged into a single median sample but insufficient regardless, supervised learning could not be considered for a classification model. Clustering visualization yielded limited insights, although it implied the isolation of sperm lineages relative to other lines in terms of expression.

**Table 2.** A random subset of the cleaned and transformed data frame of 19 469 genes and 218 Tissue_CellTypes with gene expression feature vectors represented by rows.^a^

Tissue_CellType	CKB	OR8J3	DPYS	GPR155	STX1A	CCNL1	PODN
Skeletal muscle smooth muscle cells	57.2	0.15	0.0	11.05	0.5	527.35	10.20
Tongue granulocytes	29.4	0.00	0.0	0.00	0.0	196.00	0.00
Spleen macrophages	4.1	0.00	0.0	29.50	2.0	314.20	0.00
Bone marrow plasma cells	1.5	0.00	0.0	10.80	0.0	99.90	0.00
Testis spermatocytes	193.2	0.00	0.6	0.60	1.5	43.50	0.30

^a^This would be reprocessed later with differential cluster types included in the Tissue_CellType column, which could later be parsed in BNS’ GEP component for efficient exploration.

Since supervised machine learning was not viable, proximity distancing would be considered in this work. Whilst, historically, morphological observations might have been used for cell classification, in modernity molecular insights can be used, in this case using RNA-seq data. Deviations from expression expectations can yield pathological insights, reveal therapeutic targets and perhaps even contribute to personalized, precision medicine. That said, one would need to consider normal expression limits, not to mention acquisition strategies for such data *in vivo*.

For the purposes of this study, proximity check could be achieved by matching expression feature vectors against an input vector. To reduce the impact of different scales across features, normalization would be performed. Feature ranges, deduced from columns in the full data (Table 2) were shown to vary considerably when taken across cell lines. This would justify the use of normalization. The largest range between 485,138.5 and 0.05 nTPM for the HBB (hemoglobin subunit beta) gene across cell types. Hence, Min–Max normalization would be used across each feature.

**Figure 9.**
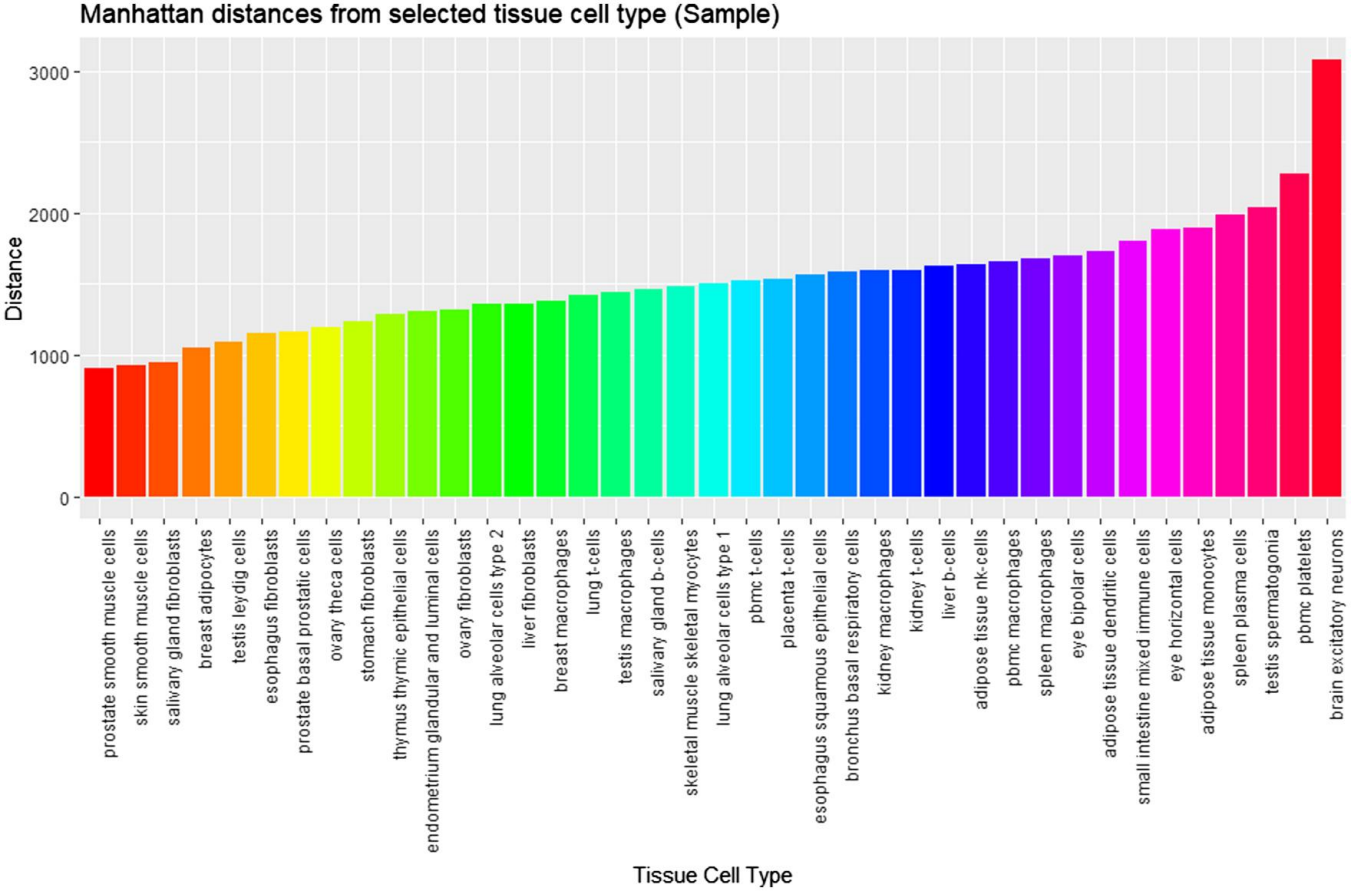
A sample of Manhattan distances from the input feature vector of adipose tissue smooth muscle cells using Min–Max normalization on features (gene expression levels). The larger the Manhattan distance, the less similar cells could be considered to be in terms of expression. It is unsurprising, therefore, that other smooth muscle cell lineages are found proximally, hence validating this statistical outcome.

Proximity metrics considered were the L1 norm (Manhattan distance) and the L2 norm (Euclidean distance). Cosine similarity might have been considered. Despite the L1 norm being considered to be more robust, there was no obvious reason that L2 could not also be considered. Nevertheless, Fig. 9 demonstrates the outcome of the use of the Manhattan distance metric based on a selected Tissue_CellType.

With the previous C# solutions only in prototype for GEP, this improved R statistical strategy could be incorporated, and a significant overhaul was undertaken under AI collaboration. In this case, phenotypic cluster variants would be used, but the overall strategy described for Manhattan distances would be retained. Using R pre-processing provided efficiency since the data would already be computed and ready to immediately display to the user. The result would be 536 tissue/cell/cluster specific expression files (843 MB), 20 082 gene specific expression files (865 MB) and a normalized Manhattan distance matrix for cell to cell comparison. All would be incorporated for ergonomic visualization in BNS. Cell to cell gene expression comparisons were considered, but it was estimated that the data would amount to 20 GB, and hence beyond deployment intentions at this time and can be considered for future updates.

### 2.8 Use of ChatGPT and productivity increase

AI, which can be considered to be fundamentally generative, has been making headway in a range of challenging domains from protein folding to mathematical operations (David *et al.* 2022, Fawzi *et al.* 2022). Indeed, in recent years generative AI within NLP has made revolutionary advancements (Vaswani *et al.* 2017, Radford *et al.* 2019, Brown *et al.* 2020, OpenAI 2023). As for its practical implementations in the software engineering of BioNexusSentinel, particularly within the specified domains of systems and synthetic Biology, we are early adopters of the existing technology as-is.

Directly integrating local LLMs in C# applications is possible via the Python communication mechanism described at the end of Section 2.2. However, for good performance this would usually require a rather powerful GPU even with smaller LLMs, and a beneficial use case would need to be designed to justify its integration. An alternative approach might be to remotely use an LLM API, although with costs considered. Thus, to avoid any misunderstanding, it should be stated that LLMs do not exist within the BioNexusSentinel software, neither was it intended to be at this early stage. ChatGPT versions were used as-is in their commercial format for their prowess in code generation, allowing for assistance in the rapid development of the software de novo through prompting the AI agent on the OpenAI website. As LLMs are approximating agents even in the best of cases (ChatGPT), LLM use would need to be carefully considered even should use cases be determined, with extensive validation of their performance against any necessary standards.

To elaborate on the practical utilization of ChatGPT, very specific algorithm design requests would be made via prompting, followed by the code generation in the language of interest. The code would be compiled and debugged via Visual Studio 2022 IDE in the case of C# or Visual Studio Code in the case of Python. Given no compilation errors, meticulous manual curation of the algorithms followed, with the option of manual alteration of the code. However, many problems could be resolved by feeding the erroneous, generated code block back to the AI agent along with details such as error codes or observed flaws as described in the English ‘natural language’. A simple example of the process of prompting and refining was the prompt ‘I want a c# winforms popup that contains the clickable links’, following the generation of the algorithm for mapping molecular species to external links.

Algorithmic methods within BNS were human written, human/machine written or machine written with human guidance and validation. Notable ChatGPT assisted implementations were the ModelSearchEngine, various conversion mappings, dialog generation and SBML syntax modifications. Perhaps the most significant was the ∼600 line PathwayMapGenerator C# code, containing the extracted SBML network structure and associating SBML standard syntax products, modifiers and reactants to reactions; along with IDs and names. Heavily ChatGPT influenced source code revolved around parsing. Parsing of data resources permitted mappings, specific data extractions, including descriptions, and model manipulations, including kinetic rate additions.

The GEP component involve the restructuring of the original dataset (rna_single_cell_type_tissue.tsv), alongside their representation with .NET controls, statistical Z-test analysis and HTTP requests. As an established statistical package R proved efficient, not in terms of ergonomics and accessibility but in terms of performance and data handling logistics. ChatGPT accelerated coding and bridged the skill gap with using R upon educated, strategical human prompting. The sentiment was that this had a dramatically beneficial impact on worker performance. It is evident that ChatGPT presented morale, productivity and exploratory benefits. That ChatGPT can provide rapid and effective answers means that a worker is rarely without highly effective council personally and technically, speaking volumes for the potential of NLP AI in LLM form.

Code developed with ChatGPT in this study required many hours of iterative refinement. Despite the extensive, iterative validation, the sense was that productivity was increased overall. It was evident that ChatGPT permitted the implementation of solutions that would likely have eluded the developer, drastically saving time and even upskilled the developer.

It pertained that the developer tracked their productivity in productive seconds of work, allowing for an analysis of productivity in the period before with the period after making acquaintance with ChatGPT. A dramatic productivity increase was found in the 50 days following ChatGPT encounter compared to the 50 days prior, confirmed by independent *t*-test. Significant *P*-values were found onwards from windows of 13 days bidirectional time (26-day period) split before and after the timestamp date, with productivity increasing to as high as 212%, with productivity subsequently sustained at 149%. This limited single sample result implied the value of intelligence support for enhancing productivity and morale, which can be provided by advanced NLP agent insights.

## 3 Discussion

BNS integrated the exploration of expression profiles with SBML manipulation and network visualizations, rendering conceptual progressions and expanding on parameterization strategies since our previous multicellular simulation research (Matzko *et al.* 2023). Specifically, by developing a resource for evaluating differential gene expression profiles locally alongside the capacity to access, download and assess a wide assortment of SBML models that could be considered for time-course simulations within the context of ongoing biophysical and biochemical solver integrations. BNS achieved this by incorporating a novel combination of libraries and bioinformatics, identifying limitations in specific bioinformatics resources (Reactome and HPA) and highlighting challenges faced towards achieving the ambitious objective of multiscale, in silico multicellular replicas, particularly with respect to biochemical models and accurate genetic representations. How such data could be transformed into the biophysical simulation domain was discussed previously in our (Matzko *et al.* 2023) and others’ (Sütterlin *et al.* 2017) work, through cellular property modulation and biomolecular changes.

BNS made an advancement in our strategies by assisting the study and quantitative handling of cytohistological expression levels, downloading and exploring of SBML models, assessment of Reactome knowledge gaps, search mechanisms related to gene expression, orthogonality of reaction network models and RNA-seq expression, as well as a prototyped automatic kinetic parameterization feature. BNS also found good use independently as a general systems biology SBML visualizer. The link to Reactome emphasized Eukarya, including Animalia, notably *Homo sapiens*, relevant for studies into medical interventions, or exploration of eukaryotic options for synthetic biology. Reactome offered a vast range of models including a developmental HOX gene model, R-I-5619507.sbml, despite its downstream truncation. Another limitation of Reactome models is a failure to describe degradation mechanics on a per model basis. Additionally, the lack of kinetics parameterization of Reactome models is a limitation that would need to be overcome with regards to using its SBML archive for time-course executions.

The expression profile analyses through RNA-seq data and bioregulatory network manipulations/exploration hence pursued the functionalization of biological data. Structural and functional laboratory investigations of multicellular systems was recommended for the in silico modelling of certain synthetic biology experiments (Naylor *et al.* 2017), which brings into question the extent that present data can allow for relevant inferences for parameterization. AlphaFold, is arguably the template in terms of validation methods for machine learned protein structural inferences in bioinformatics (Callaway 2022). Such ML inferences, or layers of inference, might assist the in silico kinetics parameterization effort from kinetics datasets such as SABIO-RK (Rojas *et al.* 2007). The biophysical interpretation of expression levels requires careful consideration, ultimately with progression towards a digital twin of biological reality. That said, the translation of expression levels from omics data (e.g. via nTPM) to accurate kinetic models remains unclear. RNA-seq itself possesses certain limitations, as RNA expression is likely no guarantee of phenotype but rather represents the transcriptome rather than the full omics of the cell. Consider the possibility of translational repression, for instance, or the possibility of expressed proteins but with RNA degraded. Further omics interactions and/or bioregulatory network investigations might shed light on these dynamic implications, and hence the integration of such additional data can be considered for expanding the research and BNS platform further. Our objective continued to be such a unified platform for multicellular CAD, which would represent information storage, analytics and functionalization. BNS represents an aspect of that wider research, serving as a complementary platform or module for further traversing and contextualizing the challenging objective of high dimensional spatiotemporal biological simulations, with future medical implications, not simply a database.

Regarding limitations of the R analysis, missing cell lineages from the dataset had an impact on Z-scores, since they are relative to the mean of the sample, so extremely rarely expressed genes might appear significant when still at trace levels. For example, the olfactory receptor family 52 subfamily H member 1 was significant in non-olfactory tissues, yet only expressed in very low amounts, without the appropriate olfactory tissue comparisons. Hence, it would also be important to ensure a more comprehensive coverage of tissue and cell types, even though the source of the expression data (HPA) was quoted as the ‘gold-standard’ in protein localization (Watson *et al.* 2022).

The internet provides a distributed environment for informatics and digital services, however with certain limitations in terms of service reliability and API consistency. Furthermore, there may be latencies, data transfer limits or service disruptions. BNS used downloaded local data and remote data. The local data provided reliability, the limitation being a loss of connectivity to updates of online resources. Community standardization can be encouraged for guaranteed access to the latest curated data for local or remote utilization. Additional resources can also be considered. For example, the NCBI (National Center for Biotechnology Information) provides gene expression data, e.g. via the Gene Expression Omnibus (NCBI 2023). Platforms such as Galaxy and ROSALIND can also be explored for omics features and tools.

BNS could be extended for drug or biosensor design, harnessing a biological model for target identification. Docking simulations associated with structural interpretations (Callaway 2022), including machine learned structural predictions, could be incorporated into an integrated CAD design tool with mechanistic foundations at the ligand level. Designing living therapeutics is another prospect, e.g. immune T-Cell therapies (BBC_News 2022) or to counteract infectious diseases using commensal bacteria (Khan *et al.* 2022), especially with a multicellular histological simulation component related to our ongoing and past (Matzko *et al.* 2023) research. From the perspective of the Z-score based methods in GEP and the R statistics for the cell lineage classifier using Manhattan distance of feature vectors, diagnostics and therapeutics might be tailored regarding the healthy range of expression norms, or indeed cell lineage classification. For the purposes of this work, the HPA (Human_Protein_Atlas 2022) was easily accessible, especially given its datasets containing cell and tissue specific expression data. rna_celline.tsv dataset from the HPA could be considered for cancer phenotypes, and there was also neurological expression data with localization. The HPA also possesses 10 million high-resolution stained cytohistological images (Digre and Lindskog 2023). The ‘secretome’ is another area of interest in the omics, and could be one source of omics data in terms of incorporating disease biomarkers. The use of the Gene Ontology or specific databases might be considered in the future to mine for specific functional components, e.g. receptors and transporters.

Stochastic simulations, unlike deterministic solutions, provide multiple trajectories, and hence behavioural distributions, via randomness and discretization (Wilkinson 2006). Deterministic rate equations, as obtained via SABIO-RK (Rojas *et al.* 2007), if requiring conversion to stochastic simulations, such as for Gillespie algorithms (Matzko *et al.* 2023), require alternative formulation. For example, COPASI 4.39 produced errors for reversible deterministic kinetics laws if simulated stochastically, requiring the decomposition into forwards and backwards steps. Automatic conversion protocols would need to be carefully elucidated before BNS integration. However, high-resolution mechanistic constants for stochastic modelling are likely to be scarce.

A capability to generate genome-scale models could be considered on the premise of associating multiple models (Büchel *et al.* 2013, Granger *et al.* 2016). BNS offered one such memory representation to work with. Construction of such networks involves mapping of genes to reactions (Dukovski *et al.* 2021), as is the case in Reactome models. Stoichiometry remains at the heart of reaction simulations, and Flux Balance Analysis (FBA) simulation methods are apparently ubiquitous in whole cell modelling at the genome scale (Weaver *et al.* 2014, Agmon *et al.* 2022). FBA avoids the need for kinetic parameters and accessible, integrated access to such a solution might be favourable also.

This work highlighted the benefits of AI in the domain of software engineering for biological CAD. It was observed that the intelligence brought by ChatGPT could increase accessibility, productivity, and functionality from the perspective of automatic programming and scripting. Albeit, requiring a level of skill, patience, prior knowledge, and rationale for effective prompt engineering and software integration, alongside debugging and algorithmic function validation. Although somewhat beyond the scope of this work, the integration of LLMs inside BNS could also be possible, including with finetuning for specific domains. However, the use case would need to be carefully considered, along with technical know-how and sufficient computational resources. AI agents such as ChatGPT are fundamentally sequence generation tools trained on vast corpora, and can be used both for training fine-tuned input–output text-to-text sequence generation, or in the case of this paper as-is for text-to-text conversion from prompt/code to code/information. This specific capability has most obvious use in data mining and automatic scripting. However from the experience of developing BNS there is no reason to assume that manual validation would not be required for meeting specific use cases, whether training data was curated in the past or present. Indeed, often in machine learning the careful curation of datasets is one of the most arduous aspects. That said, an example target for ML related to phenotypic modulation might be allele variant data via gnomAD, for instance in behavioural phenomics (Rosenhahn *et al.* 2022). The input–output modalities or multimodalities would be at the discretion of the researcher for the desired and intended informational transformations, assuming that the data is suitable. Indeed, this present work itself was restricted to using Manhattan distances for classification and relative expression comparison due to the limited number of samples in the otherwise extensive RNA-seq profiles, hence prohibiting supervised learning with neural networks due to the impossibility of formulating a train-validation-test split. That said, upon visiting other resources (e.g. gnomAD), BNS’s cytohistological expression profiler immediately becomes useful as a source of genetic profiles and metadata through gene symbols that can then be considered for further exploration in other tools, easily launched locally from a taskbar.

There are medical implications for biological CAD. Multicellular simulations and bioinformatics software solutions such as BNS can be seen as potential platforms towards an as yet undefined more comprehensive system towards spatially considered physiological reset from pathological or undesirable physical states. BNS represents a step forward in terms of accessible bioinformatics that can be used in this regard, including in terms of calculating distances from expectation with the Cell Lineage Classifier system. In addition, it represents an outstanding educational tool.

Software solutions, whether universal integrated solutions or coding modules, often benefit from incremental enhancements over sustained periods of time (e.g. operating systems over decades). Hence, regarding such updates: subgraph pruning would be useful for species (not only reactions), filtering strategies in NetworkTools could allow the easier identification of a species’ reaction involvements, aesthetics of the interface might be improved in terms of font scales and clipped regions (expandable panels might be possible), improved model organizer and metadata display, increased interactivity between the network and expression modules, validation of non-orthogonal model matches (for example instant focus on the species of interest), the possible integration of LLMs for analysing and contrasting descriptions, and the reassessment of parsing mechanisms to validate the intelligent universality of SBML loading. An interesting use of LLMs would be to task them with navigating and/or interpreting network structural associations, but just as a lack of downstream modelling limits potential use in simulations, such a strategy would be restricted to the limitations of the network models. Naturally, this might be coupled with data from GEP. In fact, LLMs could be tasked with extending network models into a desired format, such as the BNS memory model, by mining information banks or literature. A technically challenging possibility would be to analyse the true biological impact of elevated expression levels, which might differ depending on other system parameters, in addition to the integration of a wider assortment of omics, which can be treated in the effective pre-processed manner found in BNS. A ChangeLog.txt is available on the GitHub.

## Data Availability

BioNexusSentinel project releases, with corresponding data and installation instructions, are available at https://github.com/RichardMatzko/BioNexusSentinel.
